# Mercury accumulation efficiency of different biomonitors in indoor environments: the case study of the *Central Italian Herbarium* (Florence, Italy)

**DOI:** 10.1007/s11356-023-31105-3

**Published:** 2023-11-24

**Authors:** Francesco Ciani, Silvia Fornasaro, Renato Benesperi, Elisabetta Bianchi, Jacopo Cabassi, Luca Di Nuzzo, Lisa Grifoni, Stefania Venturi, Pilario Costagliola, Valentina Rimondi

**Affiliations:** 1 Department of Earth Science, University of Florence, Via G. La Pira 4, 50121 Florence, Italy; 2 Department of Earth Science, University of Pisa, Via Santa Maria 53, 56126 Pisa, Italy; 3 Department of Biology, University of Florence, Via G. La Pira 4, 50121 Florence, Italy; 4 Institute of Geosciences and Earth Resources (IGG), National Research Council of Italy (CNR), Via G. La Pira 4, 50121 Florence, Italy

**Keywords:** Mercury, Herbarium, Barks, Lichens, Mosses, Biomonitoring

## Abstract

Biomonitoring studies are often employed to track airborne pollutants both in outdoor and indoor environments. In this study, the mercury (Hg) sorption by three biomonitors, i.e., *Pinus nigra* bark, *Pseudovernia furfuracea* lichen, and *Hypnum cupressiforme* moss, was investigated in controlled (indoor) conditions. In comparison to outdoor environments, controlled conditions offer the opportunity to investigate more in detail the variables (humidity, temperature, pollutants speciation, etc.) that control Hg uptake. The biomonitors were exposed in two distinct periods of the year for 2 and 12 months respectively, in the *halls* of the *Central Italian Herbarium* (Natural History Museum of the University of Florence, Italy), which are polluted by Hg, due to past plant sample treatments. The Hg sorption trend was monitored every 3 weeks by recording: (i) the Hg content in the substrata, (ii) gaseous elemental mercury (GEM) concentrations in the exposition halls, (iii) temperature, (iv) humidity, and (v) particulate matter (PM) concentrations. At the end of the experiment, Hg concentrations in the biomonitors range from 1130 ± 201 to 293 ± 45 μg kg^−1^ (max–min) in barks, from 3470 ± 571 to 648 ± 40 μg kg^−1^ in lichens, and from 3052 ± 483 to 750 ± 127 μg kg^−1^ in mosses. All the biomonitors showed the highest Hg accumulation after the first 3 weeks of exposure. Mercury concentrations increased over time showing a continuous accumulation during the experiments. The biomonitors demonstrated different Hg accumulation trends in response to GEM concentrations and to the different climatic conditions (temperature and humidity) of the *Herbarium halls*. Barks strictly reflected the gaseous Hg pollution, while lichen and moss accumulation was also influenced by the climatic conditions of the indoor environment. Mercury bound to PM seemed to provide a negligible contribution to the biomonitors final uptake.

**Supplementary Information:**

The online version contains supplementary material available at 10.1007/s11356-023-31105-3.

## Introduction

Environmental monitoring and quantification of potentially toxic elements is often carried out by instrumental devices (Mikkelsen et al. 2005; Cabassi et al. 2017; Rimondi et al. 2022). Biomonitoring, i.e., the use of organisms as biomonitors to track changes in the environment and monitor air quality (Conti 2008; Friberg et al. 2011; Aničić Urošević and Milićević 2020; Lattanzi et al. 2020), is an alternative/complementary technique to support and integrate data from instrumental devices. Biological monitoring traditionally applies in outdoor environments. Here, it has the main advantages of being specific, efficient, and low cost, allowing air quality monitoring even in remote areas (Szczepaniak and Biziuk 2003) thanks to the permanent and natural occurrence of the organisms suitable for biomonitoring. Among all biological species, mosses and lichens are widely and commonly used in biomonitoring because: (i) they are easily recognizable; (ii) they are widely distributed even in polluted areas, proving elevated tolerance; (iii) they are characterized by slow growth and longevity; (iv) their morphology does not vary following seasonality; (v) they provide sufficient materials for numerous sampling and analysis; (vi) they are suitable to be transplanted in polluted areas to perform active biomonitoring (Bargagli 2016). In the last years, several studies pointed out the possibility to also use plant portions as biomonitors, like tree barks or leaves (Kuang et al. 2007; Tomaševič et al. 2008; Cocozza et al. 2016; Viso et al. 2021). Tree barks are likely very efficient for the accumulation and retention of atmospheric substances, in particular mercury (Hg), thanks to their structural porosity and the absence of metabolic processes (Schulz et al. 1999; Chiarantini et al. 2016; Costagliola et al. 2017; Rimondi et al. 2020; Viso et al. 2021).

In the last years, several studies have dealt with indoor biomonitoring of heavy metals, mainly using lichen and mosses (Canha et al. 2014; Protano et al. 2017; Capozzi et al. 2019; Sorrentino et al. 2021; Sujetovienė and Česynaitė 2021). The growing interest in this topic is linked to the large amount of time that people spend in indoor environments, like households and workplaces (Jones 1999; WHO 2006). Here, the air quality is even worse than outside due to the presence of multiple pollution sources (Vardoulakis et al. 2020), like pesticides, paints, and batteries (Zwozdziak et al. 2013; Jha et al. 2020).

Mercury is a global pollutant, ubiquitously distributed in the environment and naturally occurring in the Earth’s crust (Fitzgerald and Lamborg 2003; Driscoll et al. 2013). In the air, Hg occurs both in gaseous forms, i.e., elemental gaseous Hg (gaseous elemental mercury (GEM) or Hg^0^) and reactive gaseous Hg (RGM or Hg^2+^), and as particulate-bounded Hg (PBM) (Selin 2009; Weiss-Penzias et al. 2015). Despite the increasing efforts to reduce this environmental pollutant, as ratified by the UN Minamata Convention on Mercury (UNEP 2013), atmospheric Hg concentrations can occur at dangerous levels in indoor environments (Loupa et al. 2017). Indoor atmospheric Hg pollution in residential settings is mainly linked to materials that contain Hg salts as additives, like paints, cleansers, and home medications, or can be found as Hg^0^ in some household devices, like thermometers, fluorescent light bulbs, or gas flow meters (Carpi and Chen 2001). However, the main exposures to airborne Hg for humans are in workplace activities, especially industrial facilities like coal-fired power stations, metal extraction, waste incineration, and chemical industries, or the Hg use as amalgam in the dentistry sector (Pandey et al. 2011; Khwaja et al. 2016; Kolipinski et al. 2020; Ciani et al. 2021a).

Here, we present the results obtained performing biomonitoring experiments in an indoor environment with particular characteristics such as a museum botanical section, i.e., a herbarium. These museum sections are often affected by serious indoor Hg pollution, due to the past use of corrosive sublimate (HgCl_2_), employed to prevent plant infestation (Briggs et al. 1983; Hawks et al. 2004; Oyarzun et al. 2007; Kataeva et al. 2009; Fellowes et al. 2011; Webber et al. 2011; Havermans et al. 2015; Fallon et al. 2016; Marcotte et al. 2017; Cabassi et al. 2020; Ciani et al. 2021b). Three different biomonitors were used, tree barks, lichens, and mosses, simultaneously exposed in indoor atmosphere of the *Central Italian Herbarium* (Natural History Museum, University of Florence, Italy), to monitor the Hg pollution that affects this museum section. The study aimed to (i) test the Hg accumulation efficiency of the different biomonitors, (ii) verify if they reflect the indoor Hg concentrations, and (iii) get insights into the mechanisms governing Hg accumulation. Based on our knowledge, this is the first time that three different biomonitors were exposed at the same time in an indoor environment. Biomonitoring studies carried out in indoor settings where environmental (i.e., climatic) conditions are controlled (temperature, humidity, etc.) are fundamental to get insights on the organisms’ bioaccumulation capacity and mechanisms.

## Materials and methods

### Study site: indoor conditions and working scheme

**Fig. 1:**
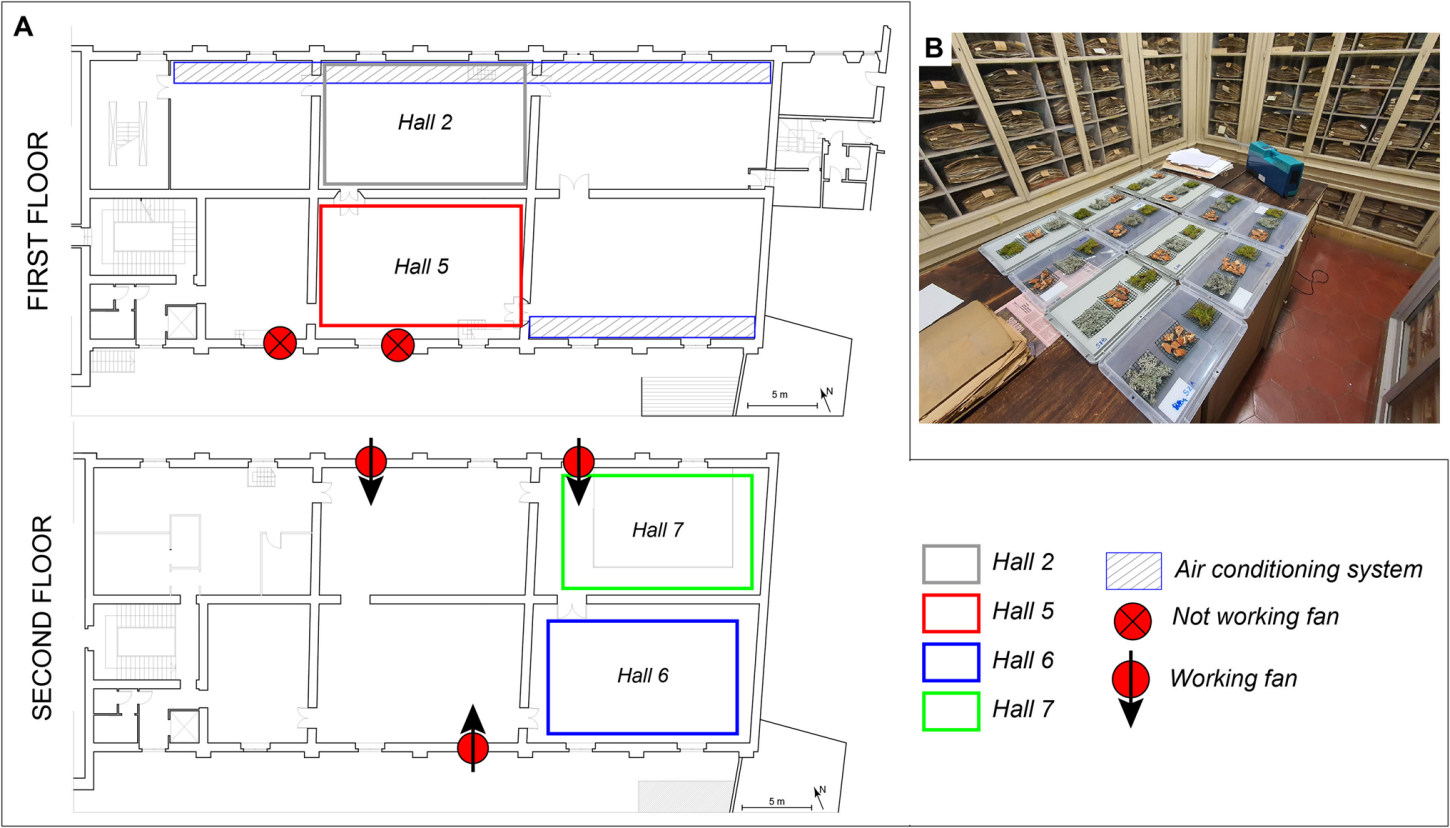
The *Central Italian Herbarium* composed by two floors with their distinctive climatic conditions (**A**); examples of biomonitors exposure before the beginning of the E1 and associated Lumex 915 M location for GEM measurements (**B**)

The *Central Italian Herbarium* is one of the largest botanical collections worldwide, hosting about 5 million plant samples (Moggi 2009; Thiers 2018). Mercury dichloride was employed from the *Herbarium*’s foundation (1842) until the beginning of the last century (Passerini and Pampanini 1927). Recent studies proved the presence of high Hg concentrations in all the exhibition rooms, both as GEM and PBM (Cabassi et al. 2020; Ciani et al. 2021b). The *Herbarium* is located on two different floors of the same building (Fig. 1a), which show distinctive features in terms of both Hg concentrations and climatic conditions. The first floor is characterized by year-round homogeneous Hg concentrations (Cabassi et al. 2020). In fact, almost all the rooms are equipped with an air conditioning system, with no air exchange with the outside, which activates at night (from 07.00 P.M. to 07.00 A.M.) (Fig. 1a). The second floor is not climatized (except for one room), but an air ventilation system consisting of some window fans ensures an air exchange with the outside (Fig. 1a). The fans are daily activated from 03.00 to 08.00 A.M, and they are located in all the *Herbarium* rooms except *hall 6*, also named *Webb Hall*. The latter, hosting the most ancient and precious collections of the museum, is the hotspot of Hg contamination especially in summer, when GEM concentrations > 50 μg m^−3^ were recorded (Cabassi et al. 2020).

To test the biomonitoring substrata in the most different conditions of exposure (Hg concentrations, ventilation, air conditioning), four rooms (two for each floor) were selected for the experiment. At the first floor, we selected *hall 2* and *hall 5*, which roughly have the same Hg concentrations (Cabassi et al. 2020), but are distinctive for the climatizing conditions (*hall 2*: air-conditioned, *hall 5*: not air-conditioned). At the second floor, we selected *hall 6* (the *Webb Hall*), for its high GEM concentrations and no air exchange with the outside, and *hall 7*, where GEM concentrations were lower (Cabassi et al. 2020) and where a window fan is daily switched on (Fig. 1a).

In these four *Herbarium halls*, the biomonitors were exposed in two distinct periods of the year and at different times. In the first period (experiment 1, E1), the samples were exposed for 6 weeks from middle April 2021 to the end of May 2021, in order to test the efficiency of the biomonitors to accumulate Hg. In the second period (experiment 2, E2), the plant samples were exposed for 12 months, from August 2021 to August 2022.

### Biomonitor sampling and analysis

The three biomonitors were sampled from a remote area in the Appennino Pistoiese (44°07′39″N, 10°40′33″E, 1,239 m., Cutigliano, Italy) far from known pollution sources. *Pinus nigra* J.F.Arnold barks were sampled directly from the trunk trees at about 1.5 m above ground level; lichens (*Pseudovernia furfuracea* L.) and mosses (*Hypnum cupressiforme* Hedw.) were collected from the bark of fir trees at approximately 1 m from the ground to prevent soil contamination (Giordani et al. 2020). As recommended in Cecconi et al. (2019), all the samples were put in plastic bags. In the laboratory, the samples were cleaned removing (i) the external layer for the barks and (ii) the residues of soil, animals, or other plants for lichens and mosses. As required by the internal *Herbarium* protocol, the biomonitors were then stored at − 20 °C up to the beginning of the experiments and then exposed in pre-cleaned plastic trays on the *halls*’ desks daily used by the *Herbarium* workers (Fig. 1b). Three exposure points were set up in all the *halls*, each with a tray (20 × 30 cm) containing the same mass of bark (5 g in E1, 10 g in E2), lichen (3 g in E1, 5 g in E2), and moss (2 g in E1, 4 g in E2). The biomonitors were sampled before the start of the experiments (time zero, T0) and every 3 weeks (T3, T6, T9, and so on) for a total of 6 and 18 weeks for E1 (April 2021 to the end of May 2021) and E2 (from August 2021 to November 2021), respectively. For E2, samples were also collected after a whole year of exposure (E2-TY, August 2022).

Every 3 weeks, each sampling involved the removal of the following quantities of material from each biomonitors: 1 g of the outer bark layer of *P. nigra* (2–3 mm), being generally the part with more accumulation capacity of the tree bark (Loppi et al. 1997; Savas et al. 2021; Bardelli et al. 2022; Isinkaralar 2022); 0.5 g of the marginal parts of the *P. furfuracea* laciniae (up to 2.5 cm from lobe tips), as generally applied in lichen biomonitoring (Giordani et al. 2020); 0.3 g of the photosynthesizing green part of *H. cupressiforme*, following the moss monitoring protocol (ICP 2015). Sampled materials were stored in paper bags and in air-dried conditions until the analysis. Once in the laboratory, fragments of retrieved transplanted materials were homogenized with a ceramic mortar and pestle up to pulverization to reach enough amount of material for analysis.

Mercury concentrations (*C*_Hg_, μg kg^−1^) were measured on 0.02–0.1 g of material using a tri-cell direct Hg analyzer (Milestone DMA-80 evo, Department of Earth Sciences, University of Florence): the instrument allows to estimate Hg concentrations in the range 0.0003–1500 ng. Analysis accuracy was tested at the beginning and at the end of each analytical run using international standards (pine needle NIST SRM 1575a, Hg = 39.9 ± 0.7 μg kg^−1^; tomato leaf NIST SRM 1573a- tomato leaf, Hg = 34.1 ± 1.5 μg kg^−1^; lake sediment BCR-280R, Hg = 1460 ± 200 μg kg^−1^), with an error within 10% (recovery percentages 92–98%). Each sample was analyzed in triplicate from each exposure point (three for each *hall*), and RSD was < 15%. Since the results were consistent between each *hall*, *C*_Hg_ for each bioindicator were reported as mean values for each room ± SD. Mercury concentrations were corrected considering the dry weight of each bioindicator, determined as reported in Chiarantini et al. (2016).

Mercury data were also reported as consecutive accumulation percentage (Ac, %), i.e., the accumulation recorded every 3 weeks of exposure, calculated as:

$$Ac\left(\%\right)=\frac{{C}_{\mathrm{Hg} }{T}_{X}-{C}_{\mathrm{Hg} }{T}_{x-1}}{{C}_{\mathrm{Hg} }{T}_{x-1}}*100$$

where *C*_Hg_*T*_*x*_ refers to the *C*_Hg_ of each bioindicator at sampling time T3, T6, etc., while *C*_Hg_*T*_*X*−1_ refers to *C*_Hg_ at the previous exposition time.

At the end of both experiments (E1 and E2), the final accumulation percentage (Af%) was calculated as

$$Af\left(\%\right)=\frac{{C}_{\mathrm{Hg} }{T}_{f}-{C}_{\mathrm{Hg} }T0}{{C}_{\mathrm{Hg} }T0}*100$$

where *C*_Hg_*T*_*f*_ refers to the *C*_Hg_ of each bioindicator at the end of the experiments, while *C*_Hg_*T0* refers to *C*_Hg_ before exposition (T0).

### GEM, PM, and indoor climate records

Gaseous elemental mercury concentrations (μg m^−3^) were recorded during the experiments using a Lumex® RA-915 M analyzer (see Cabassi et al. (2022) for instrument specification and principle of operation). Measurements were carried out approximately once a week for at least 24 h (1 measure per minute) just next (10–30 cm) to the biomonitoring species. Accuracy analysis of the Lumex was tested before the start of each measurement using its self-testing autocalibration method, assuming an acceptable error within 20%, as suggested by the manufacturer himself.

In the exposition *halls*, average temperatures (T_A_) during E1 and E2 were recorded by the Lumex® analyzer. Before the start of E2 (July 2021), a 24-h survey of climatic parameters such as relative humidity (RH), indoor temperature (T), and particulate matter concentrations (PM2.5 and PM10) was made in all the exposition *halls* using a SDS011 (Nova Fitness Co., CN) PM sensor, equipped with a DHT22 temperature and relative humidity sensor and a Real Time Clock RTC DS3231 module. Such sensors were connected through an Arduino Uno Rev3 microcontroller to a MicroSD Card Breakout Board for data logging with a 1-min acquisition frequency. During these records, GEM concentrations were also measured using the Lumex® analyzer.

### Statistical analysis

The differences of biomonitors *C*_Hg_ recorded during both experiments (E1 and E2) were investigated using the non-parametric Mann–Whitney test, due to the non-normal distribution of data. The test was performed by comparing the *C*_Hg_ reached by all the biomonitors at each sampling time with the previous exposition time (i.e., T6 vs T3, T9 vs T6). The analysis was carried out using R-Studio software (R Core Team 2018) with a significance level equal to 0.05 for all procedures. At the end of E2, scatter plot graphs were drawn to compare Hg accumulation among the different biomonitors during both E1 and E2. Dot plots were divided by color according to the exposition *halls* and their GEM concentrations. The analysis and the graphical elaborations were made using the ggpubr package with the R-Studio software (R Core Team 2018).

## Results

### Barks

**Fig. 2:**
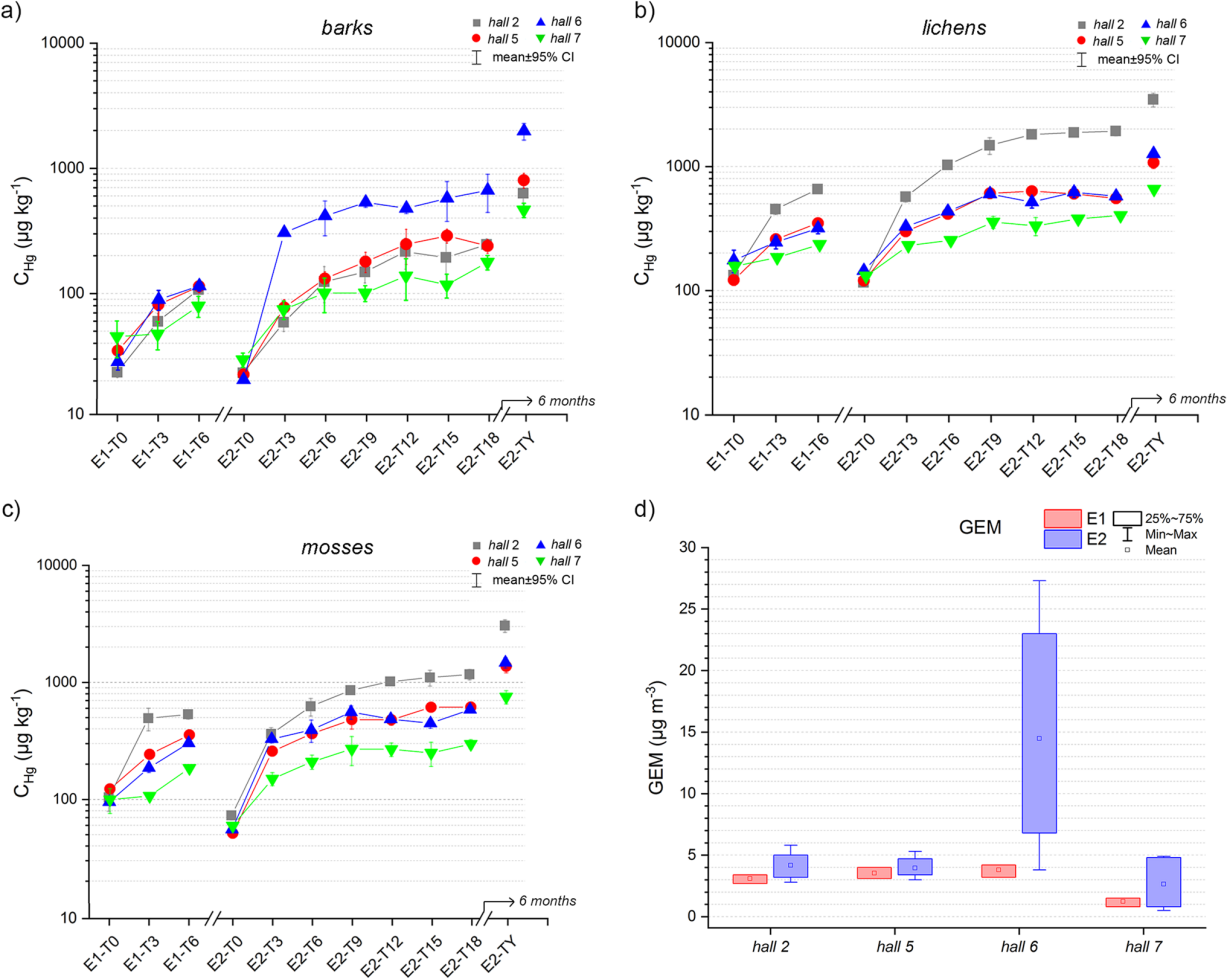
*C*_Hg_ (μg kg^−1^) in barks (**a**), lichens (**b**), and mosses (**c**) during the two experiments (E1, E2), and the GEM values (μg m^−3^) recorded during the experiments in the investigated halls (**d**)

*P. nigra* barks exposed in the different *Herbarium halls* during the E1 showed initial (E1-T0) *C*_Hg_ varying between 24 ± 2 μg kg^−1^ (min, *hall 2*) and 37 ± 10 μg kg^−1^ (max, *hall 7*) (Table 1; Fig. 2, Table S1). During all 6 weeks of the E1, the *C*_Hg_ significantly increased (Mann–Whitney test *p* < 0.05, Table S1). After the first 3 weeks of exposure (E1-T3), the maximum *C*_Hg_ was displayed in *hall 6* (*C*_Hg_ = 70 ± 15 μg kg^−1^), while the minimum was recorded in *hall 7* (*C*_Hg_ = 38 ± 11 μg kg^−1^). At the end of E1 (E1-T6), the highest final net *C*_Hg_ (i.e., the difference of *C*_Hg_ in T6-T0) was reached in *hall 2* (*C*_Hg_ = 83 ± 16 μg kg^−1^), while the lowest in *hall 7* (*C*_Hg_ = 24 ± 13 μg kg^−1^).

**Table 1:** Results of the two experiments (E1, E2); *C*_Hg_ in barks, lichens, and mosses (mean ± SD, μg kg^−1^)

		E1-T0	E1-T3	E1-T6	E2-T0	E2-T3	E2-T6	E2-T9	E2-T12	E2-T15	E2-T18	E2-TY
Barks	hall 2	24 ± 2	60 ± 10	107 ± 15	23 ± 2	59 ± 10	124 ± 40	149 ± 30	216 ± 60	194 ± 80	246 ± 30	630 ± 140
	hall 5	31 ± 7	67 ± 20	90 ± 10	21 ± 4	63 ± 10	102 ± 20	136 ± 30	181 ± 70	208 ± 30	176 ± 30	523 ± 80
	hall 6	25 ± 4	70 ± 15	87 ± 10	18 ± 1	211 ± 15	278 ± 110	348 ± 40	315 ± 30	372 ± 160	425 ± 170	1130 ± 200
	hall 7	37 ± 10	38 ± 10	61 ± 10	25 ± 4	57 ± 10	75 ± 30	75 ± 10	99 ± 40	86 ± 20	124 ± 35	293 ± 45
Lichens	hall 2	133 ± 20	453 ± 60	655 ± 30	116 ± 10	570 ± 70	1030 ± 90	1481 ± 300	1815 ± 120	1878 ± 60	1928 ± 200	3470 ± 570
	hall 5	123 ± 15	261 ± 20	353 ± 10	121 ± 10	303 ± 20	419 ± 30	615 ± 30	639 ± 65	609 ± 40	559 ± 70	1087 ± 150
	hall 6	178 ± 50	249 ± 40	323 ± 40	147 ± 10	333 ± 10	442 ± 35	610 ± 50	524 ± 70	631 ± 80	585 ± 40	1286 ± 70
	hall 7	155 ± 20	182 ± 10	232 ± 20	129 ± 5	228 ± 10	251 ± 10	352 ± 50	327 ± 70	374 ± 20	398 ± 35	648 ± 40
Mosses	hall 2	104 ± 30	494 ± 140	533 ± 60	73 ± 10	363 ± 60	623 ± 140	858 ± 90	1016 ± 100	1101 ± 220	1172 ± 160	3052 ± 480
	hall 5	121 ± 10	239 ± 26	351 ± 20	51 ± 5	254 ± 20	359 ± 30	473 ± 100	471 ± 60	604 ± 40	606 ± 60	1350 ± 200
	hall 6	95 ± 10	186 ± 22	303 ± 20	55 ± 2	328 ± 30	391 ± 110	556 ± 95	485 ± 45	446 ± 50	584 ± 50	1474 ± 190
	hall 7	100 ± 30	107 ± 10	185 ± 10	59 ± 40	150 ± 30	209 ± 40	269 ± 90	268 ± 45	249 ± 80	296 ± 40	750 ± 130

The Ac after 3 weeks of exposure (E1-T3) was between + 3% (*hall 7*) to + 189% (*hall 6*), while it decreased in E1-T6 (+ 33 to + 87%; Table S1). At the end of the E1, the Af reached the maximum value (+ 357%) in *hall 2* and the minimum value (+ 65%) in *hall 7* (Table S1).

During the E2, barks showed initial (E2-T0) *C*_Hg_ varying between 18 ± 1 μg kg^−1^ (min, *hall 6*) and 25 ± 4 μg kg^−1^ (max, *hall 7*) (Table 1; Fig. 2, Table S1). As observed for E1, barks *C*_Hg_ increased significantly (Mann–Whitney test *p* < 0.05, Table S1) in the first 6 weeks (E2-T6, Table S1). After the first 3 weeks of exposure (E2-T3), the maximum *C*_Hg_ was recorded in *hall 6* (*C*_Hg_ = 211 ± 15 μg kg^−1^), whilst the minimum in *hall 7* (*C*_Hg_ = 57 ± 10 μg kg^−1^). In the next 3 weeks (E2-T6), the *C*_Hg_ accumulated decreased, as observed for E1. At E2-T18, *hall 6* barks showed the highest *C*_Hg_ (425 ± 170 μg kg^−1^) and a final net *C*_Hg_ (T18-T0) of 407 ± 107 μg kg^−1^, while the lowest *C*_Hg_ was recorded in *hall 7* (124 ± 35 μg kg^−1^), with a final net *C*_Hg_ of 99 ± 20 μg kg^−1^.

The highest Ac was recorded at T3 in all exposition *halls* (mean value + 388%). The maximum Ac was reached in *hall 6* (+ 1047%), the minimum in *hall 7* (+ 134%). After 6 weeks of exposure (E2-T6), Ac decreased (mean value + 61%, + 33% and + 28% in *hall 6* and *hall 7*, respectively). During the following exposure times, the Ac was quite stable (ca. + 30%, mean value), with the exception at the E2-T15 (+ 6%). At the end of E2, the highest Af was shown in *hall 6* (+ 2207%), the lowest in *hall 7* (+ 396%) (Table S1).

After 1 year of exposure (E2-TY), the barks *C*_Hg_ significantly increased (Mann–Whitney test *p* < 0.05, Table S1): the highest *C*_Hg_ was observed in *hall 6* (*C*_Hg_ = 1130 ± 200 μg kg^−1^, Af =  + 6038%), the lowest in *hall 7* (*C*_Hg_ = 293 ± 45 μg kg^−1^, Af =  + 1070%). The mean *C*_Hg_ reached by the barks of all exposition *halls* was 644 ± 353 μg kg^−1^ (Af =  + 2843%).

### Lichens

*P. furfuracea* thalli employed in the E1 showed initial *C*_Hg_ varying between 123 ± 15 μg kg^−1^ (min, *hall 5*) and 178 ± 50 μg kg^−1^ (max, *hall 6*) (Table 1, Fig. 2, Table S2). After the first 3 weeks of exposure (E1-T3), the maximum *C*_Hg_ was recorded in *hall 2* (*C*_Hg_ = 453 ± 60 μg kg^−1^), the minimum in *hall 7* (*C*_Hg_ = 182 ± 10 μg kg^−1^). The highest *C*_Hg_ at the end of E1 (E1-T6) was recorded in *hall 2* (*C*_Hg_ = 655 ± 30 μg kg^−1^), the lowest in *hall 7* (*C*_Hg_ = 232 ± 20 μg kg^−1^). The *C*_Hg_ increase was significant during the entire E1 (Mann–Whitney test *p* < 0.05, Table S2).

The Ac after 3 weeks of exposure (E1-T3) varied among + 20% (*hall 7*) and + 252% (*hall 2*), while it sensibly decreased in E1-T2 (Table S2). At the end of E1, the Af reached the maximum value (+ 394%) in *hall 2* and the minimum value (+ 50%) in *hall 7* (Table S2).

During E2, the lichens thalli showed initial *C*_Hg_ varying from 116 ± 10 (*hall 2*) to 147 ± 10 μg kg^−1^ (*hall 6*) (Table 1). The lichens Hg accumulation was significant during the first 9 weeks of E2 (Mann–Whitney test *p* < 0.05, Table S2). After the first 3 weeks of exposure (E2-T3; Table S2), the maximum *C*_Hg_ was recorded by the lichens exposed in *hall 2* (*C*_Hg_ = 570 ± 70 μg kg^−1^), the minimum by those of *hall 7* (*C*_Hg_ = 228 ± 10 μg kg^−1^). At the end of E2 (E2-T18), the highest net *C*_Hg_ was recorded by the samples of *hall 2* (*C*_Hg_ = 1,812 ± 200 μg kg^−1^), the lowest was reached by the lichens in *hall 7* (*C*_Hg_ = 268 ± 40 μg kg^−1^).

The greatest Ac was reached everywhere after only 3 weeks (+ 187%, mean value of all *halls*): the maximum value corresponded to *hall 2* (+ 391%), the minimum to *hall 7* (+ 76%). The Ac decreased in the next 6 weeks (E2-T6 and E2-T9), showing a mean value of + 40% ca. (+ 81% and + 10% in *hall 2* and *hall 7*, respectively). During the following weeks, Ac sensibly reduced (mean value + 2%, + 10%, and − 1% in E2-T12, E2-T15, and E2-T18, respectively). At the end of E2, the highest Af was shown in *hall 2* (+ 1562%), the lowest in *hall 7* (+ 208%; Table S2).

After 1 year of exposure (E2-TY), lichens significantly increased their *C*_Hg_ (Mann–Whitney test *p* < 0.05, Table S2): the highest *C*_Hg_ was reached in *hall 2* (*C*_Hg_ = 3,470 ± 570 μg kg^−1^), the lowest in *hall 7* (*C*_Hg_ = 648 ± 40 μg kg^−1^). The Af varied from + 401% (*hall 7*) to + 2892% (*hall 2*). The mean *C*_Hg_ reached by the lichens of all exposition *halls* was 1623 ± 1090 μg kg^−1^ (Af + 1,164%; Table S2).

### Mosses

The *H. cupressiforme* samples employed in the E1 displayed at T0 *C*_Hg_ between 95 ± 10 μg kg^−1^ (min, *hall 6*) and 121 ± 10 μg kg^−1^ (max, *hall 5*) (Table 1, Fig. 2, Tab. S3). During all the E1, their *C*_Hg_ significantly increased (Mann–Whitney test *p* < 0.05, Table S3). After 3 weeks of exposure (E1-T3), the maximum *C*_Hg_ was reached in *hall 2* (*C*_Hg_ = 494 ± 140 μg kg^−1^), the minimum in *hall 7* (*C*_Hg_ = 107 ± 10 μg kg^−1^). At the end of E1 (E1-T2), *hall 2* also showed the highest *C*_Hg_ (533 ± 60 μg kg^−1^), while the lowest was reached by the mosses in *hall 7* (*C*_Hg_ = 185 ± 6 μg kg^−1^), as already shown for lichens.

The Ac after 3 weeks of exposure (E1-T3) reached the maximum value (+ 375%) in *hall 2* and the minimum (+ 16%) in *hall 7*. Similar values were reached in *hall 5* and *6* (+ 99% and + 96%, respectively). At the end of the E1, *hall 2* showed the highest Af (+ 412%), whilst *hall 7* the lowest (+ 85%; Table S3).

The E2 started with mosses *C*_Hg_ ranging between 51 ± 5 μg kg^−1^ (*hall 5*) and 73 ± 10 μg kg^−1^ (*hall 2*) (Table 1; Fig. 2, Tab. S3).

After 3 weeks of exposure (E2-T3), the maximum *C*_Hg_ was recorded in *hall 2* (363 ± 60 μg kg^−1^), the minimum in *hall 7* (150 ± 30 μg kg^−1^). At the end of the study (E2-T18), the highest *C*_Hg_ was recorded by the mosses of *hall 2* (*C*_Hg_ = 1,172 ± 160 μg kg^−1^), while the lowest value was recorded in the samples of *hall 7* (*C*_Hg_ = 296 ± 40 μg kg^−1^). The *C*_Hg_ significantly increased (Mann–Whitney test *p* < 0.05, Table S3) during the first 9 weeks of the experiment (E6, T12).

As observed for both barks and lichens, the highest Ac was reached by mosses after the first 3 weeks (E2-T3) (+ 366%, mean value of all *halls*): the maximum value was shown in *hall 6* (+ 496%), the minimum in *hall 7* (+ 154%). Similar to the trend showed by the lichens, the Ac decreased in the next 6 weeks of exposure (E2-T6 and E2-T9), showing a mean value of + 40% ca. (+ 72% and + 19% in *hall 2* and *hall 6*, respectively). In the following 9 weeks of exposure (E2-T12, E2-T15, and E2-T18), the mean Ac was + 10% ca. At the end of the E2 (E2-T18), the highest Af was reached by the mosses exposed in *hall 2* (+ 1508%), the lowest by those of *hall 7* (+ 400%; Table S3).

At the end of the 1-year exposition (E2-TY), the *C*_Hg_ was again significant (Mann–Whitney test *p* < 0.05, Table S3): mosses showed the highest *C*_Hg_ in *hall 2* (*C*_Hg_ = 3052 ± 480 μg kg^−1^, Af + 4086%), the lowest in the *hall 7* (*C*_Hg_ 750 ± 130 μg kg^−1^, Af + 1164%). The mean *C*_Hg_ reached by mosses of all exposition *halls* was 1656 ± 160 μg kg^−1^ (Af + 2680%).

### GEM and indoor climatic condition records

The GEM concentrations and average temperatures (T_A_) recorded during E1 and E2 in the *Herbarium halls* are reported in Fig. 2 and Table S4.

During E1, the *Herbarium halls* of the first floor displayed similar GEM concentration, with mean values at the end of the E1 of 3.1 ± 0.4 μg m^−3^ in *hall 2* (2.7–3.4 μg m^−3^, min–max) and of 3.5 ± 0.4 μg m^−3^ in *hall 5* (3.1–4.0 μg m^−3^). At the second floor, *hall 6* showed a mean GEM value of 3.8 ± 0.5 μg m^−3^ (3.2–4.2 μg m^−3^), higher than that of 1.2 ± 0.4 μg m^−3^ (0.8–1.5 μg m^−3^) displayed in *hall 7*. The *T*_*A*_ were similar in the *halls* of the first floor (mean 19.5 °C and 20.6 °C in *hall 2* and *5*, respectively), while at the second floor, the mean temperatures were slightly lower (ca. 18 °C both in *hall 6* and *7*).

During E2, mean GEM concentrations were higher than during E1 at both floors, as expected considering that E2 was performed during summertime when, as a consequence of temperature increase, GEM increases in all the *halls* (Cabassi et al. 2020). However, it showed remarkable differences among the two floors. At the climatized first floor, the investigated *halls* showed homogeneous GEM concentrations of 4.2 ± 1 μg m^−3^ and 3.9 ± 1.0 μg m^−3^ at *hall 2* and *hall 5*, respectively. On the contrary, GEM concentrations at the second floor, which is not climatized, were extremely inhomogeneous, spanning one order of magnitude of difference between *hall 6* (3.8–27.3 μg m^−3^; mean 14.5 ± 8 μg m^−3^) and *hall 7* (0.5–4.9 μg m^−3^; mean 2.6 ± 2 μg m^−3^). GEM concentrations seemed to follow the same trend of the *T*_*A*_ recorded during E2: the mean *T* values recorded on the first floor were still comparable with E1 in both *halls* (mean 19.6 °C and 20.8 °C in *hall 2* and *5*, respectively), while higher temperatures were recorded at second floor (mean 26.1 °C and 23.5 °C respectively in *hall 6* and *7*), with values close to 30 °C in the first month of exposure (August 2021) (Table S4).

To evaluate climatic data, a 24-h-long survey of relative humidity (*RH*), indoor temperature (*T*), particulate matter (PM2.5, PM10), and GEM was carried out before the start of E2 (Fig. S1 and Table S5). At the first floor, when the air conditioning system was switched on (07.00 P.M.–07.00 A.M. ca.), the air-conditioned room (*hall 2*) showed a *RH* increase of about 15% (from 40.1 to 54.6%), and a *T* decrease of about 6 °C (from 25.3 to 19.4 °C). In the not air-conditioned room (*hall 5*), a lower decrease of both *RH* (about 7%) and *T* (around 3 °C) was observed. Regarding PM and GEM, the activation of the air conditioning system sensibly reduced PM (− 300% and − 700% for PM2.5 and PM10, respectively) in both *halls*, while a 10 to 25% increase in GEM was observed (from about 7 to 7.6 μg m^−3^ in *hall 2*, and from 6 to 7.5 μg m^−3^ in *hall 5*). It is worth noting that the highest values of PM10 (up to 65 μg m^−3^) reached during the *hall 2* survey from about 01.00 P.M to 3.00 P.M. were ascribable to the handling of herbarium packages near the sensors (personal communication of the *Herbarium* staff).

The *RH*, *T*, and *PM* variables were only poorly affected by the presence (*hall 7*) or absence (*hall 6*) of the air ventilation system at the second floor. During ventilation in the surrounding rooms, the RH decreased in *hall 6* by about 2% (max 41.9%, min 31.8%), while it increased in *hall 7* by about 5% (max 39.2%, min 34.6%); *T* decreased of about 1 °C in both *halls*. No relevant variations were recorded for PM when the fans were turned on. The air ventilation system had instead a strong influence on GEM concentrations (Figure S1), which falls from 47 to 11.5 μg m^−3^ and from 12 to 0.5 μg m^−3^ in *hall 7* and *hall 6*, respectively. It is worth noting that the decrease was observed also where the fans are not directly present (i.e., in the *hall 6*).

## Discussion

The advantages of indoor biomonitoring compared to outdoor concerns the possibility to minimize the fluctuations of some parameters, notably GEM among them, which influence the Hg-uptake by different biomonitors. Indoor conditions, such as those encountered in the different exhibition *halls* of the *Central Italian Herbarium*, thus represented an ideal setting to study the effect of a few variables, such as contaminant (Hg) concentrations, temperature, and humidity, on the sorption capacity of organisms or plants tissue. On the other hand, the results could be extended to the outdoor environment with some caution since some conditions, such as the absence of wind, precipitation, and water leaching, may influence the Hg uptake and retention on the studied biomonitors (Szczepaniak and Biziuk 2003; Kuang et al. 2007; Adamo et al. 2007; Catinon et al. 2012).

Our results indicate that the biomonitors showed a generalized rapid and distinct Hg-uptake, with lichens and mosses showing the highest *C*_Hg_, while barks showed the lowest. As depicted in Fig. 2, the accumulation of Hg is gradual in all the biomonitors with absence of spikes, suggesting an uptake from a source where Hg is homogeneously distributed. The efficiency of indoor biomonitoring of heavy metals has already been demonstrated by several studies (Canha et al. 2014; Protano et al. 2017; Capozzi et al. 2019; Sorrentino et al. 2021; Sujetovienė and Česynaitė 2021), but few focused on Hg (Motyka et al. 2013); moreover, the use of barks as biomonitors in outdoor environment has been already stressed by several authors (Chiarantini et al. 2016; Costagliola et al. 2017), but, to the best of our knowledge, the possibility to use them in indoor settings is completely new. After only 6 weeks of exposure, all biomonitors show a significant higher amount (three times more, Mann–Whitney test *p* < 0.05) of *C*_Hg_ than the initial one, while after almost 4 months, their *C*_Hg_ is about ten times higher. Furthermore, all the biomonitors show similar Hg accumulation trends, with accumulation exceeding 200% after the first 3 weeks of exposure in all the *halls* (Ac about + 230%, mean value of all the biomonitors, Tables S1, S2 and S3 in Supplementary). These results are in line with the findings obtained in other studies in lichens (Vannini et al. 2014) and mosses (Lodenius et al. 2003), underlying the ability of these organisms to quickly retain Hg^0^ during both laboratory studies and indoor experiments. Based on our results, the same capacity can be ascribable to barks, although in this regard, no comparative indoor studies have been found in literature.

**Fig. 3:**
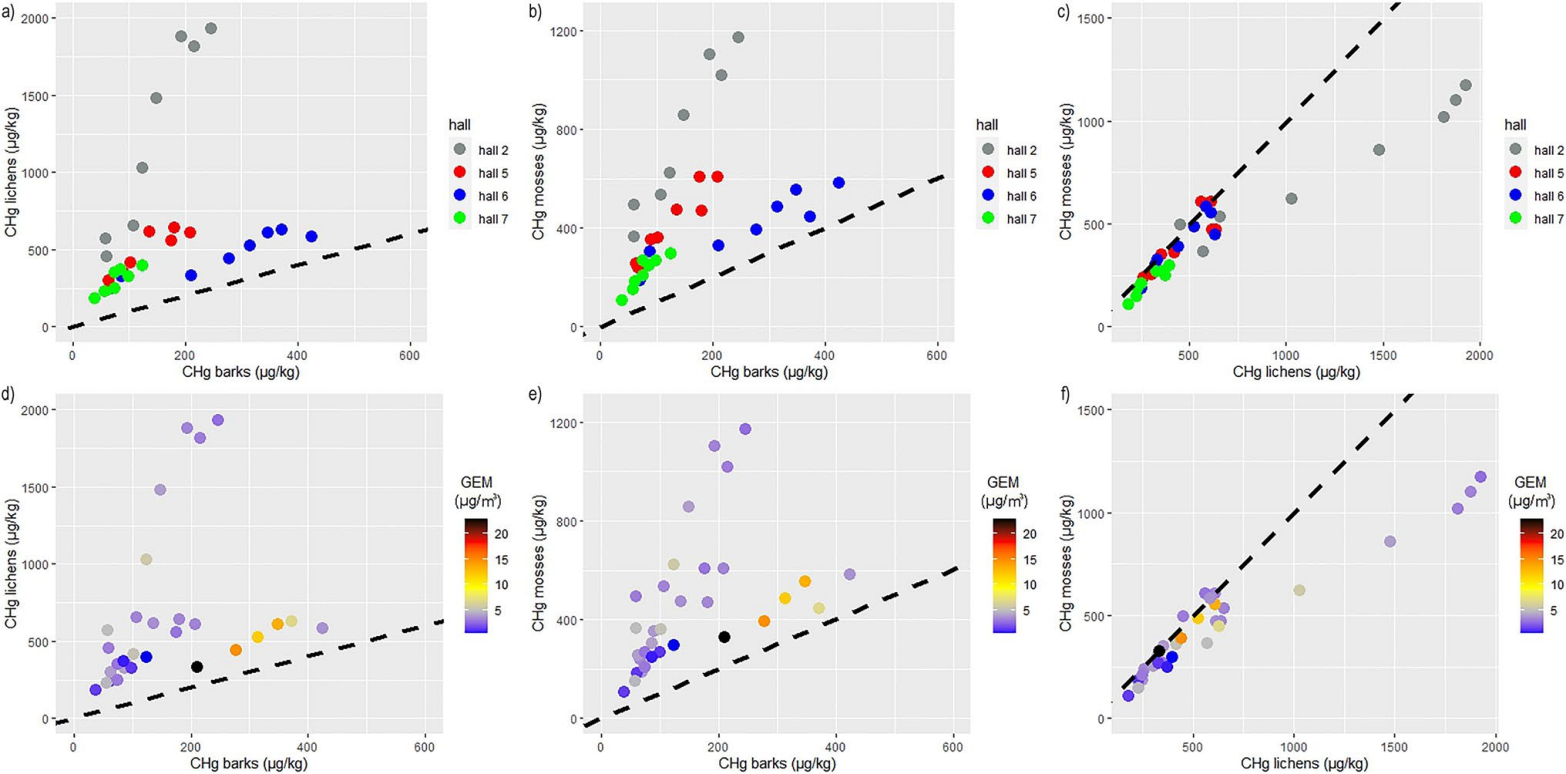
*C*_Hg_ (μg kg^−1^) in barks vs lichens (**a**), barks vs mosses (**b**), and lichens vs mosses (**c**) at the different sampling time (T1, T2) and in relation to the sampling location (*halls*); the same elaborations are reported in relation to the mean GEM concentrations (μg m^−3^) registered in the exposure time (**d**, **e**, **f**). Dashed black line refers to the hypothetical 1:1 ratio among biomonitors *C*_Hg_

The Hg-uptake of biomonitors is more evident during E2, rather than E1, due to the longer experiment duration. At the end of E1 (6 weeks), some biomonitors, notably barks, show in fact relatively small *C*_Hg_ differences between different *halls* having different GEM concentrations. Instead, these differences become more evident during the 18-week-long E2 experiment (Fig. 2). At each sampling time, *C*_Hg_ in mosses and lichens is generally four times higher than the corresponding *C*_Hg_ in barks, plotting above the 1:1 line (Fig. 3, Table S1,S2 and S3), suggesting that barks accumulate GEM less efficiently in terms of absolute concentrations. Furthermore, the results of the statistical analysis proved a significant longer Hg uptake by lichens and mosses (nine weeks, Table S2 and S3) compared to barks (6 weeks, Table S1), hinting a prolonged Hg accumulation capacity of cryptogams.

Moreover, the scatter plot graphs comparing barks vs lichens and barks vs mosses (Fig. 3 a, b, d, and e) show two distinct trends, following the peculiar climatic conditions of the *hall 2* (the air-conditioned *hall*) and the GEM concentrations reached in the *Webb Hall*. In the *hall 2*, the ratio between the *C*_Hg_ of lichens and mosses vs barks is four times higher than those recorded in the other exposition *halls*, in particular with respect to the *hall 6*, where barks demonstrate a more efficient Hg accumulation (Fig. 2). In this *hall*, tree barks also well reflect the increase in GEM from E1 to E2, a mechanism that is due to the temperature-driven volatilization processes affecting metallic Hg (Scholtz et al. 2003). The other biomonitors do not display, however, the same coherent behavior of barks with respect to GEM concentrations. Lichen and mosses during E1 and E2 reach the highest *C*_Hg_ in *hall 2*, while in *hall 5*, which showed a comparable GEM concentration, they display a distinctly lower *C*_Hg_. Coherently with the lowest GEM concentrations, all the biomonitors exposed in *hall 7* show instead the lowest *C*_Hg_.

Therefore, it is worth noting that not all biomonitors show the same behavior under different GEM concentrations and climatic conditions: this feature could be plausibly traced back to different Hg trapping processes in the different biomonitor substrates. In the case of barks, several authors (Chiarantini et al. 2016; Costagliola et al. 2017) conclude that these biomonitors essentially lack any metabolic activities, and thus, they uptake GEM via non-physiological adsorption processes. Vazquez et al. (2002) suggested that the high affinity between pine barks and bivalent cation such as Hg^2+^ could be related to the presence of tannins (procyanidin), a particular class of polyphenolic compounds involved in several plants’ reactions, including cation complexation, in woody tissues, as in angiosperms barks (Hernes and Hedges 2004). Viso et al. (2021) supposed that in *Platanus hispanica* Mill. ex Münchh. barks, gaseous Hg uptake could be regulated by the presence of lenticels, i.e., circular pores of the periderm allowing plant gas exchange. Chiarantini et al. (2017) investigated Hg speciation in pine barks and found that Hg was mainly present as a Hg-cysteine complex that certainly indicated the presence of Hg bound to thiol groups. Such authors suggested that Hg is probably adsorbed as gaseous Hg on bark surface, see also Bardelli et al. (2022), and then stably retained by thiol-containing proteins.

Differently from pine barks, as already stressed, lichens and mosses Hg content do not always strictly reflect GEM concentrations in the *halls*, while they share comparable Hg-uptake trends. More in detail, in both the experiments, they showed (i) the highest final *C*_Hg_ and Af in the air-conditioned *hall 2*; (ii) the same uptake trend in the *hall 5* and *hall 6*, which strongly differed in terms of *T*_*A*_ and GEM concentrations, especially during E2 (Fig. 2, Tab. S4); and (iii) a significant (Mann–Whitney test *p* < 0.05) Hg accumulation in the first 9 weeks of exposure during E2 (Table S2, S3). In recent years, some laboratory studies found similar Hg-uptake efficiency among lichens and mosses (Bargagli 2016, and references therein). Despite that it is commonly believed that they cannot be used interchangeably for biomonitoring purposes (Bargagli et al. 2002), Loppi et al. (1999) already found a strong correlation between Hg content in lichens and mosses sampled in a geothermal area (Mt. Amiata, Tuscany, Italy). Based on the results obtained in this study, it can be inferred that other variables than GEM affect Hg trapping by lichens and mosses. In particular, the daily effect of *T* decrease and *RH* increase as result of the air conditioning system activity in the *hall 2* (Fig. S1) produced the optimal conditions for Hg uptake by lichens and mosses. Cryptogams, contrarily to barks, are living organisms whose growth and physiology, and therefore their bioaccumulation performances, are strongly regulated by environmental variables (Du and Fang 1982; Lodenius et al. 2003; Giordano et al. 2009; Fernández et al. 2015; Zhou et al. 2021). Among them, temperature and humidity influence the permeability of their cell walls and membrane, and then the accessibility to the sites where functional groups binding cations are present (Nieboer et al. 1978). The gaseous Hg absorption of these organisms likely involves enzymatic processes, in particular a catalase activity that oxidizes Hg^0^ to Hg^2+^, a low mobility form which is progressively accumulated in plant cells thanks to the lack of a thick waxy cuticle in their epidermis (Vannini et al. 2014; Bargagli 2016). Previous studies have shown how the presence of several chemical functional groups on mosses and lichens surfaces plays a relevant role in the uptake of atmospheric contaminants (González and Pokrovsky 2014; Varela et al. 2015; Bargagli 2016). Vannini et al. (2014) showed high efficiency in Hg^0^ accumulation by *P. furfuracea*, the same lichen used in our experiments. The authors found that this species revealed a temperature-dependent uptake kinetic that reach a maximum efficiency at 20 °C, while it decreased at 30 °C. These findings could explain the high *C*_Hg_ reached by lichens in *hall 2*, where the air conditioner daily lowers the temperature to 20 °C, compared to the other *halls*. Furthermore, in the air-conditioned *hall 2*, a stronger Hg accumulation in lichens than mosses is observed (Figs. 2 and 3). This result is probably linked to the better capacity of lichens than mosses to maintain high metal-uptake performances in response to humidity variations, as observed in outdoor studies (Adamo et al. 2003; Vingiani et al. 2004).

Notably, the measurements carried out in the *Herbarium* during the present study quantified only GEM; a previous study clearly indicated the presence of particulate Hg in the *Herbarium* (Ciani et al. 2021b). In the present study, the 24-h PM surveys carried out in the four exposition *halls* (Fig. S1) indicate a general low concentration of PM and thus a negligible contribute of PBM to the total budget accumulated by the biomonitors. However, handling of plant packages by the *Herbarium* staff could occasionally increase PM, producing positive spikes of PM concentrations, as observed in the *hall 2* before the beginning of E2. The results obtained in the *hall 2* (Fig. 2) suggest that PM spikes represent an insignificant contribution to *C*_Hg_, since in this *hall* a PBM uptake, if any, is visible in lichen and mosses but not in barks. In addition, as already stressed, the accumulation curves of Fig. 2 are gradual, suggesting a Hg-uptake from a source having a homogeneous distribution of this element.

Finally, despite a stable trend that could be deduced by the flattening of *C*_Hg_ displayed especially by lichens and mosses at the end of E2, the results after 1 year of exposure (E2-TY) indicated that the biomonitors have not yet reached a saturation concentration. For example, in the *hall 7*, which displayed the lowest GEM concentration both in E1 and E2, the Hg accumulated on biomonitors tends to stabilize with time from E2-T6 onward up to E2-T18, but it is evident their increase in Hg concentration from E2-T18 to E2-TY (Fig. 2).

## Conclusions

The biomonitoring experiments carried out in the *Central Italian Herbarium* using both innovative (bark trees) or classic biomonitors (lichens and mosses) provided different information on their Hg sorption capacity.

All the biomonitors significantly demonstrated their high Hg uptake capacity after several weeks of exposure and showed similar Hg accumulation trends during the experiments, based on the conditions to which they were exposed during the experiments.

Barks displayed the lowest *C*_Hg_ among biomonitors, but the Hg-uptake on this substrate was systematically proportional to GEM concentration. Differently, lichens and mosses did not always strictly reflect GEM concentrations, but they reached the highest Hg concentrations where the variation of climatic variables (i.e., temperature and humidity) produced better conditions for their Hg-uptake.

The Hg concentrations recorded in the biomonitors probably resulted from the gaseous Hg pollution, due to the low PM concentrations found in the *Herbarium*.

All the biomonitors still continued to accumulate Hg after 1 year of exposure in the *Herbarium halls*, showing that they have not yet reached a saturation concentration.

### Supplementary Information

Below is the link to the electronic supplementary material.

Supplementary file1 (DOCX 393 KB)
