# Supplementary material for: Mercury accumulation efficiency of different biomonitors in indoor environments: the case study of the Central Italian Herbarium (Florence, Italy)
Source: Environ Sci Pollut Res Int. 2023 Nov 24;30(59):124232–44. doi: 10.1007/s11356-023-31105-3 (PMC10746580; doi:10.1007/s11356-023-31105-3)
Supplement: Supplementary file 1 — Supplementary file1 (DOCX 393 KB) [file 11356_2023_31105_MOESM1_ESM.docx]

Supplementary files for:

Mercury accumulation efficiency of different biomonitors in indoor environments: the case study of the *Central Italian Herbarium* (Florence, Italy)

Francesco Ciani, Silvia Fornasaro, Renato Benesperi, Elisabetta Bianchi, Jacopo Cabassi, Luca Di Nuzzo, Lisa Grifoni, Stefania Venturi, Pilario Costagliola, Valentina Rimondi*

*Corresponding author at: Department of Earth Science, University of Florence, Via G. La Pira 4, 50121 Firenze, Italy, Tel.: +39-055-2757506, E-mail: [valentina.rimondi@unifi.it](mailto:valentina.rimondi@unifi.it) (V. Rimondi)


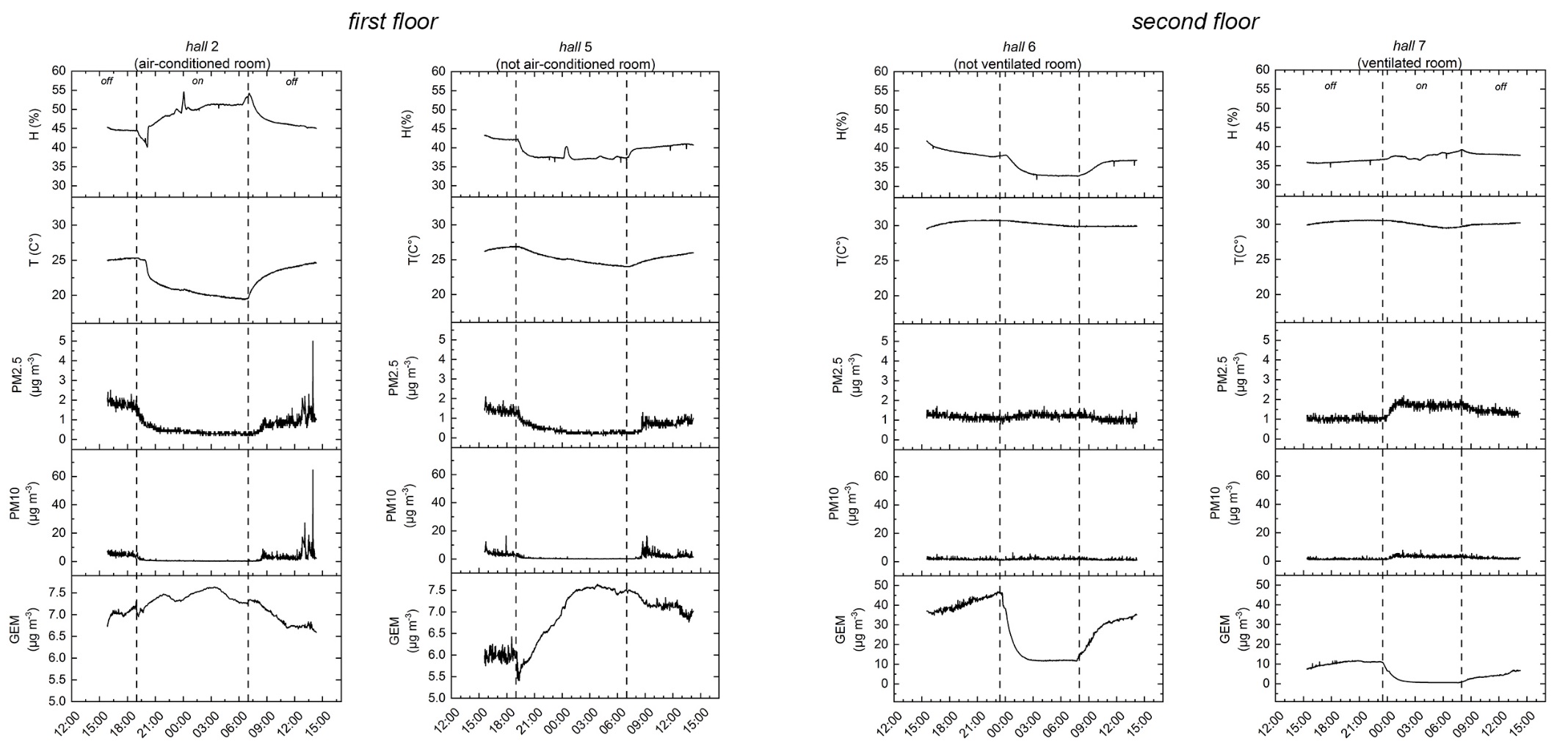


Figure S1: Results of the 24-h surveys carried out in the exposition *halls* (H= relative humidity; T= temperature; PM2.5 and PM10= particulate matter 2.5 and 10; GEM= gaseous elemental Hg).

| ***barks*** | C_Hg_ (mean±SD, μg/kg) A(%) ^a^ | | | | |
| --- | --- | --- | --- | --- | --- |
|  | *hall 2* | *hall 5* | *hall 6* | *hall 7* | *mean ^b^* |
| E1-T0 | 24±2 | 31±7 | 25±4 | 37±14 | 29±6 |
| E1-T1 | 60±10 +156% | 67±23 +128% | 70±15 +189% | 38±11 +3% | 59±14* +162% |
| E1-T2 | 107±15 +87% | 90±12 +51% | 87±9 +34% | 61±14 +33% | 86±19* +51% |
| ***net accumulation-E1***  ***(T2-T0 in E1)*** | ***84±16 +357%*** | ***59±13 +192%*** | ***62±9 +251%*** | ***24±13 +65%*** | ***57±25 +198%*** |
|  |  |  |  |  |  |
| E2-T0 | 23±2 | 21±4 | 18±1 | 25±4 | 22±3 |
| E2-T1 | 59±9 +158% | 63±12 +213% | 211±15 +1047% | 57±11 +134% | 97±75* +388% |
| E2-T2 | 124±40 +114% | 102±16 +71% | 278±111 +33% | 75±27 +28% | 145±91* +61% |
| ***net accumulation at T2-E2***  ***(T2-T0 in E2)*** | ***101±52 +439%*** | ***81±41 +386%*** | ***260±135 +1444%*** | ***50±25 +200%*** | ***123±62 +559%*** |
| E2-T3 | 149±27 +26% | 136±30 +34% | 348±36 +37% | 75±13 +9% | 177±118 +27% |
| E2-T4 | 216±60 +50% | 181±67 +43% | 315±34 -10% | 99±43 +33% | 203±89 +29% |
| E2-T5 | 194±78 -12% | 208±31 +26% | 372±155 +16% | 86±22 -5% | 215±118 +6% |
| E2-T6 | 246±27 +44% | 176±26 -13% | 425±170 +32% | 124±35 +49% | 243±131 +28% |
| ***final net accumulation-E2***  ***(T6-T0 in E2)*** | ***223±26 +959%*** | ***155±24 +743%*** | ***407±170 +2207%*** | ***99±21 +396%*** | ***221±134 +1004%*** |
| E2-TY | 630±139 +156% | 523±83 +197% | 1130±201 166% | 293±45 +136% | 644±353* +165% |
| ***net accumulation-year***  ***(TY-T0 in E2)*** | ***607±26 +2615%*** | ***502±24 +2409%*** | ***1112±170 +6038%*** | ***268±21 +1070%*** | ***622±134 +2843%*** |

Tab. S1: C_Hg_ and net accumulation (mean±SD μg/kg) recorded in barks during E1 and E2. ^a^ = A(%) determined every three weeks of exposure and at the end of the experiment; *^b^* = mean C_Hg_ in all the *halls* at the same exposition time (T). * = Mann–Whitney test *p < 0.05*.

| ***lichens*** | C_Hg_ (mean±SD, μg/kg) A (%)^a^ | | | | |
| --- | --- | --- | --- | --- | --- |
|  | *hall 2* | *hall 5* | *hall 6* | *hall 7* | *mean^b^* |
| E1-T0 | 133±20 | 123±15 | 178±48 | 155±17 | 147±25 |
| E1-T1 | 453±56 +252% | 261±19 +116% | 249±38 +47% | 182±8 +20% | 286±116* +108% |
| E1-T2 | 655±27 +47% | 353±13 +35% | 323±40 +30% | 232±19 +27% | 391±184* +35% |
| ***final net accumulation-E1***  ***(T2-T0 in E1)*** | ***523±21 +394%*** | ***230±11 +187%*** | ***145±54 +81%*** | ***77±29 +50%*** | ***243±196 +165%*** |
|  |  |  |  |  |  |
| E2-T0 | 116±6 | 121±7 | 147±14 | 129±4 | 128±12 |
| E2-T1 | 570±69 +391% | 303±22 +151% | 333±6 +129% | 228±6 +76% | 358±128* +187% |
| E2-T2 | 1030±92 +81% | 419±27 +39% | 442±35 +33% | 251±12 +10% | 535±294* +41% |
| ***net accumulation at T2-E2***  ***(T2-T0 in E2)*** | ***914±457 +787%*** | ***298±150 +246%*** | ***295±149 +201%*** | ***122±64 +95%*** | ***407±204 +318%*** |
| E2-T3 | 1481±295 +43% | 615±28 +48% | 610±49 +40% | 352±50 +41% | 764±427* +43% |
| E2-T4 | 1815±119 +26% | 639±65 +4% | 524±71 -14% | 327±71 -7% | 827±581 +2% |
| E2-T5 | 1878±57 +4% | 609±40 -4% | 631±79 +21% | 374±19 +20% | 873±589 +10% |
| E2-T6 | 1928±201 +3% | 559±66 -8% | 585±37 -6% | 398±35 +6% | 867±617 -1% |
| ***final net accumulation-E2***  ***(T6-T0)*** | ***1812±196 +1562%*** | ***438±68 +361%*** | ***438±39 +299%*** | ***268±37 +208%*** | ***739±623 +577%*** |
| E2-TY | 3470±571 +80% | 1087±146 +94% | 1286±74 +120% | 648±40 +63% | 1623±1091* +87% |
| ***net accumulation-year***  ***(TY-T0)*** | ***3354±566 +2892%*** | ***966±147 +797%*** | ***1139±80 +776%*** | ***519±40 +401%*** | ***1495±1097 +1164%*** |

Tab. S2: C_Hg_ and net accumulation (mean±SD μg/kg) recorded in barks during E1 and E2. ^a^ = A(%) determined every three weeks of exposure and at the end of the experiment; *^b^* = mean C_Hg_ in all the *halls* at the same exposition time (T). * = Mann–Whitney test *p < 0.05*.

| ***mosses*** | C_Hg_ (mean±SD, μg/kg) A(%)^a^ | | | | |
| --- | --- | --- | --- | --- | --- |
|  | *hall 2* | *hall 5* | *hall 6* | *hall 7* | *mean ^b^* |
| E1-T0 | 104±33 | 121±10 | 95±7 | 100±31 | 105±11 |
| E1-T1 | 494±140 +375% | 239±26 +99% | 186±22 +96% | 107±8 +16% | 257±167* +145% |
| E1-T2 | 533±61 +13% | 351±20 +49% | 303±24 +65% | 185±6 +74% | 343±145* +33% |
| ***final net accumulation-E1***  ***(T2-T0 in E1)*** | ***429±44 +412%*** | ***230±23 +190%*** | ***208±29 +218%*** | ***85±30 +85%*** | ***238±142 +227%*** |
|  |  |  |  |  |  |
| E2-T0 | 73±8 | 51±4 | 55±2 | 59±38 | 60±3 |
| E2-T1 | 363±62 +409% | 254±23 +404% | 328±32 +496% | 150±25 +154% | 274±18* +357% |
| E2-T2 | 623±139 +72% | 359±26 +42% | 391±111 +19% | 209±38 +41% | 396±55* +45% |
| ***net accumulation at T2-E2***  ***(T2-T0 in E2)*** | ***550±275 +753%*** | ***308±157 +604%*** | ***336±178 +611%*** | ***150±75 +254%*** | ***336±170 +560%*** |
| E2-T3 | 858±89 +46% | 473±104 +31% | 556±95 +47% | 269±97 +28% | 539±6* +36% |
| E2-T4 | 1016±97 +19% | 471±62 +4% | 485±45 -9% | 268±46 +6% | 560±24 +4% |
| E2-T5 | 1101±221 +9% | 604±42 +31% | 446±54 -7% | 249±76 -9% | 600±83 +7% |
| E2-T6 | 1172±156 +8% | 606±56 +1% | 584±48 +33% | 296±35 +35% | 665±56 +11% |
| ***final net accumulation-E2***  ***(T6-T0)*** | ***1099±149 +1508%*** | ***555±58 +1096%*** | ***528±48 +955%*** | ***237±34 +400%*** | ***605±52 +1008%*** |
| E2-TY | 3052±483 +160% | 1350±207 +123% | 1474±194 +152% | 750±127 +153% | 1656±157* +149% |
| ***net accumulation-year***  ***(TY-T0)*** | ***2979±479 +4086%*** | ***1299±210 +2563%*** | ***1418±194 +2562%*** | ***691±128 +1164%*** | ***1597±155 +2680%*** |

Tab. S3: C_Hg_ and net accumulation (mean±SD μg/kg) recorded in barks during E1 and E2. ^a^ = A(%) determined every three weeks of exposure and at the end of the experiment; *^b^* = mean C_Hg_ in all the *halls* at the same exposition time (T). * = Mann–Whitney test *p < 0.05*.

|  | **GEM** (μg/m3) | | | | | | | | | | | | |
| --- | --- | --- | --- | --- | --- | --- | --- | --- | --- | --- | --- | --- | --- |
|  | E1 | | | |  | E2 | | | | | | | |
|  | E1-T0 | E1-T3 | E1-T6 | *mean* |  | E2-T0 | E2-T3 | E2-T6 | E2-T9 | E2-T12 | E2-T15 | E2-T18 | *mean* |
|  |  |  |  |  |  |  |  |  |  |  |  |  |  |
| *hall 2* | 2.7 | 3.2 | 3.4 | *3.1* |  | 4.9 | 5 | 5.8 | 4.1 | 3.2 | 3.4 | 2.8 | *4.2* |
| *hall 5* | 3.1 | 3.5 | 4 | *3.5* |  | 4.7 | 4.1 | 5.3 | 3.7 | 3.5 | 3.4 | 3.9 | *3.9* |
| *hall 6* | 3.2 | 4 | 4.2 | *3.8* |  | 27.3 | 23 | 14.9 | 13.5 | 12.1 | 6.8 | 3.8 | *14.5* |
| *hall 7* | 0.8 | 1.5 | 1.4 | *1.2* |  | 4.9 | 4.8 | 3.1 | 3 | 1.4 | 0.8 | 0.5 | *2.6* |
|  |  |  |  |  |  |  |  |  |  |  |  |  |  |
|  | T_A_ (C°) | | | | | | | | | | | | |
|  | E1 | | | |  | E2 | | | | | | | |
|  | E1-T0 | E1-T3 | E1-T6 | *mean* |  | E2-T0 | E2-T3 | E2-T6 | E2-T9 | E2-T12 | E2-T15 | E2-T18 | *mean* |
|  |  |  |  |  |  |  |  |  |  |  |  |  |  |
| *hall 2* | 19.3 | 19.5 | 19.7 | *19.5* |  | 20.3 | 20.6 | 20.8 | 19.6 | 19.2 | 18.7 | 18 | *19.6* |
| *hall 5* | 20.6 | 20.9 | 20.4 | *20.6* |  | 21.9 | 21.6 | 22.2 | 21 | 21.5 | 19 | 18.6 | *20.8* |
| *hall 6* | 17.8 | 17.6 | 19 | *18.1* |  | 29.5 | 29.4 | 27 | 27.4 | 26.8 | 23.1 | 19.5 | *26.1* |
| *hall 7* | 16.7 | 17.2 | 20.3 | *18* |  | 26.7 | 29.2 | 26.9 | 22.4 | 21 | 20.1 | 17.8 | *23.5* |

Table S4: mean GEM concentrations (μg/m^3^) and average temperatures (T_A_) recorded during each exposure time of E1 and E2.

|  |  | **H (%)** | **T (C°)** | **PM2.5 (μg/m3)** | **PM10 (μg/m3)** | **GEM (μg/m3)** |
| --- | --- | --- | --- | --- | --- | --- |
|  |  |  |  |  |  |  |
| ***hall* 2** | *min* | 40.1 | 19.4 | 0.2 | 0.2 | 6.6 |
|  | *max* | 54.6 | 25.3 | 5.0 | 64.7 | 7.6 |
|  | *mean* | 47.9 | 22.3 | 0.8 | 2.1 | 7.2 |
|  |  |  |  |  |  |  |
| ***hall* 5** | *min* | 36.0 | 24.0 | 0.1 | 0.1 | 5.4 |
|  | *max* | 43.3 | 26.9 | 2.1 | 16.4 | 7.6 |
|  | *mean* | 39.2 | 25.3 | 0.6 | 1.6 | 6.9 |
|  |  |  |  |  |  |  |
| ***hall* 6** | *min* | 31.8 | 29.5 | 0.6 | 0.6 | 11.5 |
|  | *max* | 41.9 | 30.8 | 1.7 | 6.4 | 46.9 |
|  | *mean* | 36.2 | 30.3 | 1.2 | 1.8 | 28.0 |
|  |  |  |  |  |  |  |
| ***hall* 7** | *min* | 34.6 | 29.4 | 0.8 | 0.8 | 0.5 |
|  | *max* | 39.2 | 30.6 | 2.2 | 7.9 | 11.8 |
|  | *mean* | 37.2 | 30.1 | 1.4 | 2.3 | 5.4 |

Table S5: results of 24h surveys of relative humidity (H), temperature (T), PM2.5, PM10 and GEM made in the *Herbarium* exposure *halls*.
